# Timing is everything: Drivers of interannual variability in blue whale migration

**DOI:** 10.1038/s41598-020-64855-y

**Published:** 2020-05-07

**Authors:** Angela R. Szesciorka, Lisa T. Ballance, Ana Širović, Ally Rice, Mark D. Ohman, John A. Hildebrand, Peter J. S. Franks

**Affiliations:** 1 Scripps Institution of Oceanography, UC San Diego, 9500 Gilman Dr., La Jolla, CA 92093 US; 2 Southwest Fisheries Science Center, NOAA Fisheries Service, 8901 La Jolla Shores Dr., La Jolla, CA 92037 US; 3 Oregon State University, Marine Mammal Institute, 2030 SE Marine Science Dr., Newport, Oregon, 97365 US; 4 Texas A&M University at Galveston, 200 Seawolf Parkway, Galveston, TX 77554 US

**Keywords:** Animal migration, Behavioural ecology, Climate-change ecology, Animal behaviour

## Abstract

Blue whales need to time their migration from their breeding grounds to their feeding grounds to avoid missing peak prey abundances, but the cues they use for this are unknown. We examine migration timing (inferred from the local onset and cessation of blue whale calls recorded on seafloor-mounted hydrophones), environmental conditions (e.g., sea surface temperature anomalies and chlorophyll *a*), and prey (spring krill biomass from annual net tow surveys) during a 10 year period (2008–2017) in waters of the Southern California Region where blue whales feed in the summer. Colder sea surface temperature anomalies the previous season were correlated with greater krill biomass the following year, and earlier arrival by blue whales. Our results demonstrate a plastic response of blue whales to interannual variability and the importance of krill as a driving force behind migration timing. A decadal-scale increase in temperature due to climate change has led to blue whales extending their overall time in Southern California. By the end of our 10-year study, whales were arriving at the feeding grounds more than one month earlier, while their departure date did not change. Conservation strategies will need to account for increased anthropogenic threats resulting from longer times at the feeding grounds.

## Introduction

Productivity in the California Current Ecosystem (CCE) is fueled by the seasonal, wind-driven coastal upwelling of nutrient-rich waters^1^. Upwelling pulses are followed by phytoplankton blooms ca. one week later, and an increase in zooplankton biomass weeks to months later^2^. Seasonal upwelling and the ensuing assemblage of zooplankton and forage fish create rich feeding grounds that are exploited by highly migratory predators^3,4^. The timing of these physical-biological couplings is strongly influenced by environmental variability on interannual to multi-decadal scales^5^.

Environmental variability may create a temporal mismatch between the migration timing of a predator and fluctuations of its prey^6,7^. Migration between discrete feeding and breeding grounds involves complex internal and external processes and species-specific environmental cues^8^. At the feeding grounds, prey availability determines the timing and physical condition of an animal at its departure, which influences the timing of arrival and physical condition at its breeding grounds, ultimately affecting reproductive success^9^. Animals migrating long distances minimize predator-prey mismatches by altering the timing of their migration^10^, while balancing time spent on foraging or reproductive-related behaviors^11^. Plasticity in migration has been well studied in terrestrial birds and mammals^12,13^, but less in aquatic animals.

Blue whales (*Balaenoptera musculus*) are a model species for investigating the relationship between environmental interannual variability and migration phenology. As a long-lived, highly migratory species, individuals experience interannual to multidecadal-scale environmental variability. Although the cues they use for the timing of migration remain unknown, the Eastern North Pacific blue whale population’s general migration phenology has been well established from visual, acoustic, and tag data^14–16^. Previous studies have found that the majority of these whales occupy feeding grounds in the CCE of the United States, including waters west of the Southern California Region (SCR) from May to December before migrating south to their breeding grounds in the Costa Rica Dome (CRD) for the winter where they reproduce or give birth (Supplementary Fig. 1)^14,15,17^.

The diet of this population in the SCR is overwhelmingly dominated by two species of euphausiid krill (*Thysanoessa spinifera* and *Euphausia pacifica*)^18–20^. Blue whales are acoustically active at the feeding grounds, producing “D”, “A”, and “B” calls (Supplementary Fig. 2)^21^. D calls are downswept (~100–40 Hz) and seconds in duration. They are produced by both sexes during the months when they forage and are considered social or contact calls^22^. B calls are tonal, low-frequency (fundamental frequency <20 Hz), and long in duration (10–20 s), produced most often in repeated sequences along with A calls as part of song^23^. These songs are produced only by males and are believed to have a primarily reproductive-related function^22,23^. D calls dominate early in the year and B calls later (Supplementary Fig. 3)^24^, which together can be used as a proxy for the timing of blue whale migration. Here we test the hypothesis that the timing and drivers of migration, including the transition from predominately feeding to reproductive-related behaviors, is mediated by available prey resources and physical environmental properties.

## Results and Discussion

**Figure 1:**
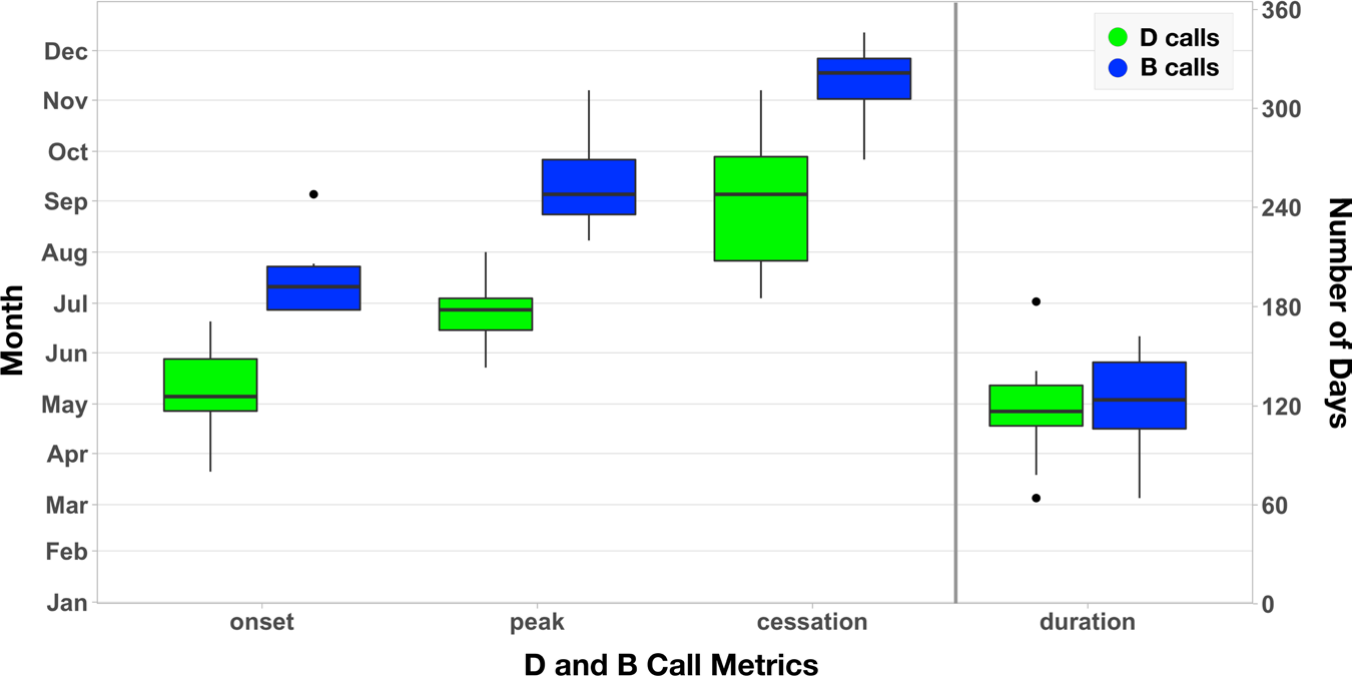
Summarized D and B call metrics for date of onset, peak, cessation, and duration (number of days). Each whisker boxplot displays the median, first and third quartiles (25th and 75th percentiles), upper and lower whiskers (1.5 times the inter-quartile range), and outliers (black circles).

The 10-year average annual cycles of D and B calls through time indicates that whales arrive at the SCR feeding grounds in May and depart in November, remaining at the feeding grounds an average of 8.4 months (Fig. 1, Supplementary Table 1, Supplementary Fig. 3). The timings of D call onset and cessation displayed greater variability than B call onset and cessation (Fig. 1), suggesting that D calls may be influenced more by external forces than B calls. There was no significant relationship between the duration of overlap of D and B calls in the same year, or between the timings of B call cessation and D call onset the following year. The onset, cessation, and duration of both D and B calls displayed interannual variability, suggesting that the timing of these calls, and, by inference, blue whale arrival and departure, was not associated with photoperiod, as has been documented for many terrestrial birds and mammals^25^. Instead, blue whales must use other cues to detect interannual variability and determine when to migrate to and from their feeding grounds.

### Arrival time at the feeding grounds is correlated with sea surface temperature anomalies from the previous feeding season

**Figure 2:**
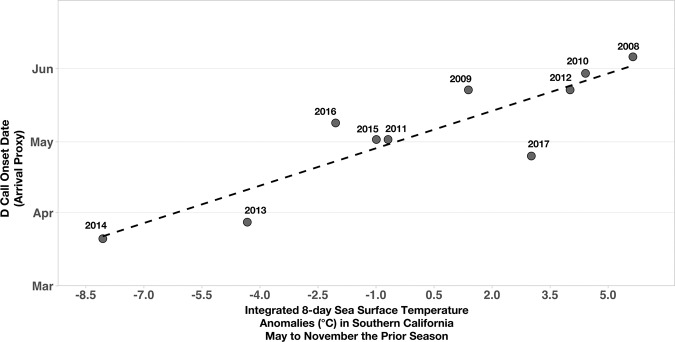
D call onset at the Southern California feeding grounds compared to integrated 8-day sea surface temperature anomalies the prior feeding season. Annual (2008–2017) onset of D calls (our proxy for blue whale arrival at the feeding grounds) correlated with integrated eight-day sea surface temperature anomalies (°C) in Southern California from the prior feeding season (May–November; multiple regression partial R^2^ = 0.78, p < 0.01).

Eight-day mean sea surface temperature (SST) anomalies integrated over May to November of the previous feeding season (the average time whales are in the SCR) were correlated with the timing of the onset of D calls (i.e., blue whale arrival) in the SCR the following year (multiple regression partial R^2^ = 0.78, p < 0.01; Fig. 2). Specifically, when the previous feeding season was colder, D call onset (i.e., arrival) in the SCR was earlier the following year, and it was later following warmer years.

Temperature is a migratory cue used by many terrestrial taxa—from insects to birds to ungulates^6,26^. In the marine realm, SST is similarly important for highly migratory species, including leatherback sea turtles (*Dermochelys coriacea*)^27^ and flounder (*Platichthys flesus*)^28^. Nishiwaki^29^ was the first to suggest that changes in SST might influence the timing of humpback whale (*Megaptera novaeangliae*) arrival on their wintering grounds. Visser *et al*.^30^ tied the timing of peak baleen whale abundance in the Azores to rising SSTs following the spring bloom, and Tsujii *et al*.^31^ found that water temperature was a good predictor of fin whale (*Balaenoptera physalus*) arrival and departure in the southern Chukchi Sea. Our study demonstrates how oceanographic conditions influence migration timing, but also suggests the use of memory.

The idea that memory of past conditions combined with resource tracking allows animals to modify the time, speed, and direction of migration movement has been documented in terrestrial mammals^32^ and birds, including demonstrating the memory of high-quality foraging locations for at least 12 months^33^. Among the limited number of studies of the timing of whale migration, to our knowledge, only one examined the role of memory. Abrahms *et al*.^34^ found that tagged blue whale latitudinal migratory movements correlated with a 10-year average spring bloom (via chlorophyll-*a* peaks) and hypothesized a long-term memory of the location of highly productive foraging sites. Our findings, and the fact that we could detect no relationship between D call onset and any environmental variable on the breeding grounds prior to arrival at the feeding grounds, support the hypothesis of memory use in migratory timing. By integrating the SST anomaly signal at the feeding grounds from the prior feeding season, whales may forecast future conditions and adjust their arrival timing the following year.

### Annual sea surface temperature anomalies from the previous season are correlated with krill biomass at the feeding grounds

**Figure 3:**
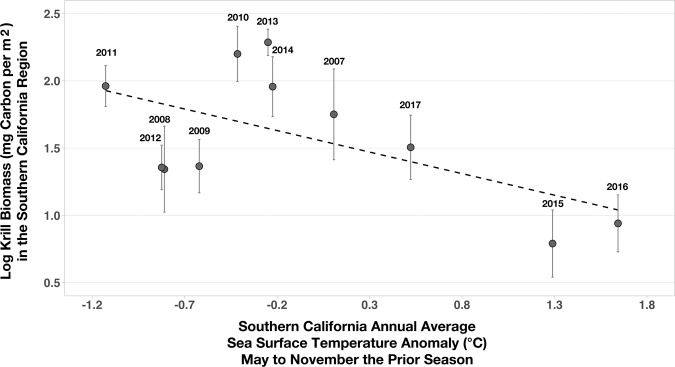
Krill biomass compared to annual sea surface temperature anomalies in Southern California the prior feeding season. Annual (2008–2017) spring biomass (log transformed; mg carbon per m^2^ with standard error bars) of adult and juvenile *Euphausia pacifica* and *Thysanoessa spinifera* correlated with annual sea surface temperature anomalies (°C) in Southern California from the prior feeding season (May-November; R^2^ = 0.43, p = 0.07).

**Figure 4:**
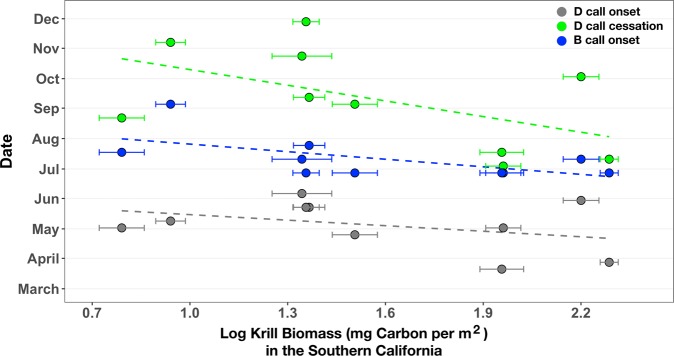
Krill biomass in the Southern California Region compared to D and B call onset and D call cessation. Annual (2008–2017) spring biomass (log transformed mg carbon per m^2^ with standard error bars) of adult and juvenile *Euphausia pacifica* and *Thysanoessa spinifera* correlated with the onset of D calls (multiple regression partial R^2^ = 0.52, p = 0.04), cessation of D calls (R^2^ = 0.28, p = 0.10), and onset of B calls (R^2^ = 0.36, p = 0.07).

Colder annual SST anomalies in the SCR the previous feeding season were associated with greater krill biomass the next year, while warmer annual SST anomalies were associated with lower krill biomass (R^2^ = 0.34, p = 0.06; Fig. 3). Previous studies have established that associations between colder water and greater zooplankton biomass result from the upwelling and advection of cold, nutrient-rich water and subsequent primary production^1^. Greater krill biomass in the SCR was also associated with an earlier onset of D calls, when whales arrived at their feeding grounds (multiple regression partial R^2^ = 0.52, p = 0.04; Fig. 4). The relationship between SST anomalies, krill biomass, and D call onset suggest that in addition to anticipating future conditions based on the prior year’s conditions, whales could profitably use SST as a proxy for krill biomass. Thus, whales could optimize arrival time at the feeding grounds to take advantage of abundant prey (cold years, early arrival) or limit their effort in the area when they expect prey to be impoverished (warm years, late arrival).

Analyses of blue whale scat collected in the SCR have shown that whales preferentially target *T. spinifera*^20^, which has a greater lipid content than *E. pacifica*^35^, though both euphausiid species compose their diet and are dominant in the cold waters of the SCR^36^. Zooplankton in cold, high-latitude waters have higher energy contents and lipid stores than zooplankton at lower latitudes^37^. Given the potential for a greater caloric value from cold-water compared to warm-water euphausiid prey, there may be a significant feeding advantage for whales to arrive on their feeding grounds earlier when it is colder, and when lipid-rich krill is predictably more abundant.

During warmer years, it is possible that whales delay departure from the CRD to opportunistically feed locally on smaller, less energy-rich krill such as *Euphausia eximia*, *Euphausia gibboides*, *Euphausia distinguenda*, *Nematoscelis gracilis*, *Nematobrachion flexipes*, and *Nyctiphanes simplex* before migrating to the SCR^38,39^. However, while some data suggest that whales may feed in the CRD^15^, there is no evidence that the bulk of this population remains year round or that the euphausiid species available at blue whale breeding grounds^40^ could support the energetic demands of the population. Another possibility, suggested from analyses of visual, acoustic, and satellite tagging data^15,40,41^, is that in warm years blue whales leave the CRD, but stop in the Gulf of California and along Baja California Peninsula, where they feed opportunistically on the subtropical euphausiid *Nyctiphanes simplex* on their way to a relatively less-productive SCR.

### Krill biomass mediated the transition to reproductive-related calling behavior at the feeding grounds

Higher krill biomass in the SCR was associated with earlier D call (multiple regression partial R^2^ = 0.52, p = 0.04; Fig. 4) and B call (R^2^ = 0.36, p = 0.07; Fig. 4) onset there, as well as earlier D call cessation (R^2^ = 0.28, p = 0.10; Fig. 4). In years when whales had access to greater-than-average krill biomass, they ceased D calls sooner and started to produce B calls sooner. Also, in years with greater krill biomass, the duration of D calling was shorter, though not significantly (R^2^ = 0.23, p = 0.16). The production of song by male humpback whales has been studied extensively, including on feeding grounds^42,43^, and proposed hypotheses regarding the purpose of these songs include intersexual and intrasexual functions^44^. While the production of B calls by male blue whales is likely associated with reproduction, their calling behavior is complex and, like humpback whale song, the precise functions are unknown^22^. However, our discovery of the link between higher krill biomass at the feeding grounds and blue whales’ earlier transition to reproductive-related calling behavior (i.e., onset of B calls) reinforces the hypothesized importance of the connections among migration timing, prey quality, and reproductive-related behavior^25,45^.

### The time of arrival of blue whales to the feeding grounds showed long-term trends

**Figure 5:**
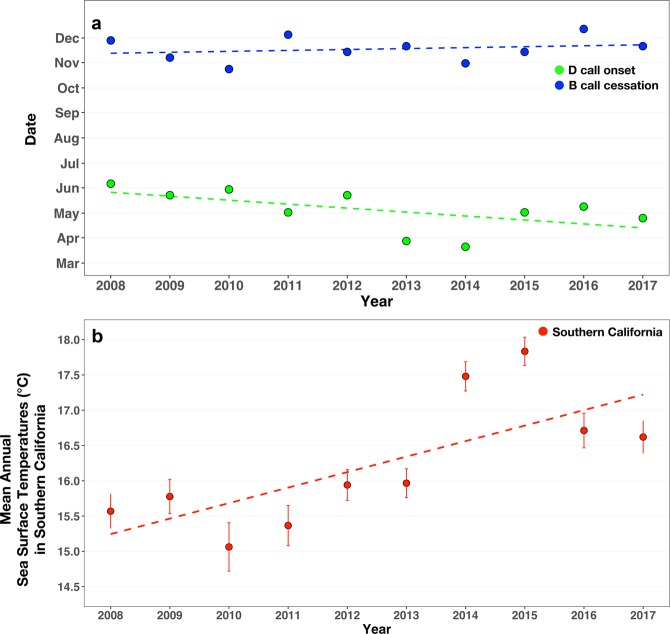
Long-term trends in D call onset, B call cessation, and annual average sea surface temperatures. (**a**) D call onset date (i.e., arrival at the feeding grounds) shifted significantly earlier across the 10-year study period (2008–2017; multiple regression partial R^2^ = 0.53, p = 0.04) while there was no significant shift in b call cessation (i.e., departure from the feeding grounds) (R^2^ = 0.05, p = 0.53). (**b**) Across the same time period, there was a significant increase in average annual sea surface temperatures (°C with standard error bars) in Southern California (R^2^ = 0.55, p = 0.01).

The onset of D calls (our proxy for blue whale arrival at the SCR feeding grounds), showed a long-term trend of earlier onset over the 10-year period (multiple regression partial R^2^ = 0.53, p = 0.04), shifting more than one month (42 days) from June to April. Across the same 10-year period, mean annual SST in the SCR increased by 1 °C (R^2^ = 0.55, p = 0.01; Fig. 5b). We hypothesize that the decadal warming trends are driving the whales to arrive at the SCR feeding grounds earlier. This has led to whales spending more and more time at the SCR feeding grounds.

Long-term change in migration timing has been demonstrated in amphibians, birds, insects, fish, marine invertebrates, marine zooplankton, and mammals^6,46^. There are few studies of long-term temporal changes in migration timing for highly migratory aquatic animals; however, the continued collection of photographic identification, passive acoustic monitoring, and satellite tag data, is revealing early arrival trends in other whale species. Across a 27-year period, fin and humpback whales arrived one month earlier on their feeding grounds in the Gulf of St. Lawrence in the North Atlantic Ocean. Because this timing shift was significantly correlated with increased sea-surface temperature and decreased sea-ice formation, it was hypothesized that the shift in arrival allowed whales to track changes in the timing of the spring bloom^47^. A similar study using telemetry and acoustic data found that over a 22-year period, beluga whales (*Delphinapterus leucas*) in the Eastern Beaufort Sea departed later in years with delayed sea-ice freeze-up, which likely enhanced productivity and zooplankton advection^48^.

Our findings show that blue whales have altered their timing of migration in the CCE of the United States. We hypothesize that as the waters in the SCR and CRD are warming, the quality and quantity of krill biomass are changing, removing any previous advantage of remaining longer in the CRD or along the coast of Baja to feed on smaller, less energy-rich species of krill. Krill biomass in the SCB also shows a long-term increase^49^, suggesting an advantage for blue whales spending more time at the feeding grounds off the California coast. In addition to changes in krill biomass, previous studies have documented krill range contractions, and range shifts coincident with physical oceanographic changes^50^, which may further influence blue whale migration behavior.

These long-term adjustments to changes in prey distribution and availability may result in whales following their prey poleward, remaining on feeding grounds longer, or suspending migration. Bioenergetic models indicate that increases in travel time resulting from poleward expansion increase the overall energetic cost of migration and reduce the time available for feeding, reproduction, and calving^51^. A longer migration or feeding period may result in a decreased frequency of migration, especially if the cost of migration becomes too high^51,52^. Any adjustments by the whales to track changes in prey distribution and biomass may also increase their spatial overlap with anthropogenic threats, further threatening this already endangered species. For example, in the case of this population, because of the high volume of ship traffic in the SCR, increased residence time could increase the whales’ lifetime risk of being struck by a ship^53^.

### The time of departure of blue whales from the feeding grounds shows long-term stability

The cessation of B calls, our proxy for blue whale departure from the feeding grounds did not change across the 10-year study period (R^2^ = 0.05, p = 0.53, Fig. 5a). In other mammals, migration phenology has been shown to be influenced by a combination of external biotic and abiotic cues, as well as by endogenous biological clocks regulating the physiological and morphological changes necessary for these behaviors^6^. Hormones linked to migration timing include melatonin^6^, adipose and thyroid hormones, and gonadal steroids^54^. Although virtually unstudied in the context of migration for marine mammals, these hormones likely play a role in their transition from feeding to reproductive-related calling, possibly triggering whale migration back to their breeding grounds. Because the cessation of B calls was not related to any environmental indices or krill biomass at the feeding grounds, we hypothesize that the cessation of B calls, our proxy for departure from the feeding grounds, may be partially regulated by seasonal fluctuations in hormone levels.

Leptin, which has been studied in bowhead whales (*Balaena mysticetus*) and beluga whales^55^ is a satiety hormone that is released as adipose tissue increases, signaling to the reproductive system that sufficient fuel reserves have been stored to support reproduction^25^ and stimulating ovulation in female mammals^56^. Additional evidence of the influence of hormones comes from the analysis of cross-sections of baleen from a stranded male blue whale, which displayed regularly spaced areas of high testosterone peaks^57^. Although the age of the whale was unknown, the cycles mirrored annual cycles of testosterone measured in the baleen of a bowhead whale and North Atlantic right whale (*Eubalaena glacialis*) of known ages^57^. Seasonal fluctuations in testosterone have also been measured from blubber samples of male humpback whales with mean testosterone peaks between November and January^58,59^. The similarity in annual testosterone cycles for these baleen whale species and similarities between humpback and blue whale reproduction support the idea that hormones play a role in migration phenology, especially triggering departure back to the CRD breeding grounds.

## Conclusions

The timing of blue whale arrival at their feeding grounds and start of reproductive calling behavior appears to be driven by an interaction with temperature and prey. We hypothesize that in addition to real-time perceptual cues, blue whales use the memory of the previous year’s integrated SST anomalies at the feeding grounds as an indicator of next year’s krill biomass and to time their arrival at the SCR feeding grounds. Fluctuations in krill biomass were not only correlated with D call onset (our proxy for blue whale arrival at the feeding grounds), but also with the timings of the cessation of D calls and the onset of B calls at the SCR feeding grounds. These relationships suggest that krill — in particular lipid-rich, cold-water krill biomass — is an important driver of the timings of migration and the start of reproductive calling behavior. The phenotypic plasticity exhibited by blue whales has apparently allowed them to accommodate interannual variability while balancing these biological imperatives. However, despite the interannual variability in arrival time, a long-term trend emerged from our data showing that although blue whales departed at the same time each year, they arrived at their summer feeding grounds more than one month earlier by the end of our 10-year study. There may come a time when adjustments in timing without geographic displacement will not be sufficient to allow both feeding and reproduction during a single year. These whales may be forced to follow their prey poleward, to remain on feeding grounds longer, or to suspend migration, with potential costs to the time available for mating and reproduction. Long-term changes in the migration phenology of endangered blue whales present pressing conservation and management issues, such as possible increases in the spatial or temporal overlap of the whales with commercial ships, fishing gear, and other anthropogenic threats.

## Methods

### Acoustic data collection and processing

Acoustic data were collected from 2008 to 2017 at five sites in the SCR using seafloor-mounted high-frequency acoustic recording packages (HARPs; Supplementary Fig. 4, Supplementary Table 2)^60^. The data were decimated by a factor of 100 to create an effective acoustic bandwidth from 10 to 1,000 Hz (for data sampled at 200 kHz) or 10 to 1,600 Hz (for data sampled at 320 kHz). Long-term spectral averages with 5-s temporal and 1-Hz frequency resolution were created for each deployment using the custom software package Triton in MATLAB^61^. A modified version of the generalized power-law detector^62^ was used to automatically detect blue whale D calls. All D call detections were manually verified, and false detections were removed from subsequent analyses. Blue whale B calls were automatically detected using the spectrogram cross-correlation method^63^, described by Širović *et al*.^64^. All B call detections from February to May (when blue whale calls are scarce) were verified by a human analyst to remove false detections. Because D and B calls are generally temporally offset (Supplementary Fig. 3), together they encompass the majority of the period when whales are present in the SCR and calling.

### Call and migration metric calculations

We multiplied the daily number of calls by the fraction of daily sampling time to correct for any partial recording effort. We then pooled calls into weekly median bins and normalized the calls to be between 0 and 1 by scaling with the maximum number of daily calls per year. Annual cycles were defined as February through January to ensure late calls from one migration cycle did not get counted in the beginning of another. Four migration timing metrics were defined per call type: onset, peak, cessation, and duration. Because there can be low levels of calls recorded year-round, the onset and cessation of each call type were calculated as thresholds that encompassed 90% of the total number of calls relative to the day with the peak number of calls (Supplementary Fig. 5, Supplementary Table 1). Thus, onset and cessation specified the start and end of the bulk of calls, respectively. Duration was calculated as the number of days from onset to cessation (Supplementary Table 1). The onset of D calls and cessation of B calls in each year were used as proxies for arrival and departure date, respectively.

### Environmental indices

Call metrics were compared with environmental indices of various spatial and temporal scales (Supplementary Table 3). Basin-wide Pacific Ocean environmental indices included the monthly North Pacific Gyre Oscillation (NPGO) index and the monthly Pacific Decadal Oscillation (PDO) index as indicators of overall productivity. The equatorial-specific index included the Oceanic Niño Index (ONI), a 3-month running mean of ERSST.v5 SST anomalies in the Niño 3.4 region (5°N-5°S, 120–170°W) as an indicator of El Niño and La Niña events. Regional 8-day environmental indices included area-averaged SST (4 microns, night only; °C) and chlorophyll *a* (mg/m^3^) in the SCR 32–35°N, 121–117°W; Supplementary Fig. 1) and CRD (5–15°N, 100–85°W; Supplementary Fig. 1) derived from MODIS-Aqua level-3 data as proxies for conditions in those regions. Additional SCR environmental indices included the cumulative upwelling index (CUI; 33°N, 119°W), which was calculated from integrated mean daily upwelling indices, and spring adult and juvenile *E. pacifica* and *T. spinifera* biomass, the preferred prey of Eastern North Pacific blue whales in the SCR^18–20^. Spring krill biomass data (mg carbon per m^2^) were retrieved from the Brinton and Townsend Euphausiid Database, converted to organic C biomass from relationships described in Lavaniegos & Ohman^65^. All samples were collected with a 0.71-m diameter, 0.505 mm mesh bongo net towed from 210–0 m during California Cooperative Oceanic Fisheries Investigations (CalCOFI) cruises (Supplementary Table 4). Sample processing was conducted by the Ohman lab and the Scripps Institution of Oceanography Pelagic Invertebrate Collection. Annual spring biomass data were averaged from CalCOFI lines 80 to 93 and stations 26 to 60 (Supplementary Fig. 6) and log (x + 1) transformed. Only nighttime tow samples were included in the calculations to account for diel vertical migration and net avoidance^66^.

### Statistical analyses

Because D and B call metrics were not cross correlated, they were investigated as independent response variables. To investigate environmental cues that could influence when whales leave the CRD or before they arrive in the SCR, we first examined individual linear regressions of D and B call metrics and environmental indices (with seasonal cycles removed) at various lagged durations to investigate the potential explanatory power of each environmental index. Only D and B call onset, peak, and cessation were used because durations did not correspond to specific dates, and durations were correlated with their respective call cessation dates. Environmental indices with high coefficient of determination (R^2^) or goodness of fit were then used in forward and backward selection stepwise multiple regression using the ‘MASS’ package in R to examine the relationships between significant environmental indices and call metrics. Only one model required multiple regression (Supplementary Table 5); the remaining models presented were individual linear regressions. Model selection was based on Akaike’s Information Criterion^67^. The partial R^2^ for each variable in the multiple regression was determined using the ‘rsq’ package in R. Significance level was set at 0.10, in order to minimize the probability of Type II errors in studies with limited sample sizes^68^. Recording effort varied over time and at each site per call (Supplementary Fig. 7; Supplementary Methods and Analyses).

## Supplementary information

Supplementary Information.

Supplementary Information.

Supplementary Information.

Supplementary Information.

Supplementary Information.

Supplementary Information.

Supplementary Information.

Supplementary Information.

## Data Availability

The datasets generated for this study are available upon request to the corresponding author.

## References

[1] Huyer A (1983). Coastal upwelling in the California Current System. Prog. Oceanogr..

[2] Chenillat F, Rivière P, Capet X, Franks PJ, Blanke B (2013). California coastal upwelling onset variability: cross-shore and bottom-up propagation in the planktonic ecosystem. PLoS One.

[3] Block BA (2011). Tracking apex marine predator movements in a dynamic ocean. Nature.

[4] Batchelder HP (2013). Climate impacts on zooplankton population dynamics in coastal marine ecosystems. Oceanography.

[5] Kudela RM (2008). New insights into the controls and mechanisms of plankton productivity in coastal upwelling waters of the northern California Current System. Oceanography.

[6] Visser ME, te Marvelde L, Lof ME (2012). Adaptive phenological mismatches of birds and their food in a warming world. J. Ornithol..

[7] Lennox RJ (2016). Conservation physiology of animal migration. Conserv. Physiol..

[8] Dingle H, Drake VA (2007). What is migration?. BioScience.

[9] Harrison XA, Blount JD, Inger R, Norris DR, Bearhop S (2011). Carry-over effects as drivers of fitness differences in animals. J. Animal Ecol.

[10] Walther GR (2002). Ecological response to recent climate change. Nature.

[11] Perrin N, Sibly RM (1993). Dynamic models of energy allocation and investment. Annu. Rev. Ecol. Evol. Syst..

[12] Lehikoinen E, Sparks TH, Zalakevicius M (2004). Arrival and departures dates. Adv. Ecol. Res..

[13] Sparks TH (2007). How consistent are trends in arrival (and departure) dates of migrant birds in the UK?. J Ornithol..

[14] Calambokidis J (1990). Sightings and movements of blue whales off central California 1986–88 from photo‐identification of individuals. Rep. int. Whaling Comm. (Special Issue).

[15] Mate B, Lagerquist B, Calambokidis J (1999). Movements of North Pacific blue whales during the feeding season off Southern California and their southern fall migration. Mar Mam Sci.

[16] Burtenshaw JC (2004). Acoustic and satellite remote sensing of blue whale seasonality and habitat in the northeast Pacific. Deep Sea Res..

[17] Širović A (2015). Seven years of blue and fin whale call abundance in the Southern California Bight. Endang Species Res.

[18] Fiedler P (1998). Blue whale habitat and prey in the California Channel Islands. Deep Sea Res..

[19] Croll D (2005). From wind to whales: trophic links in a coastal upwelling system. Mar. Ecol. Prog. Ser..

[20] Nickels CF, Sala LM, Ohman MD (2018). The morphology of euphausiid mandibles used to assess predation by blue whales in the southern sector of the California current system. J. Crustac. Biol.

[21] McDonald M, Hildebrand JA, Mesnick SL (2006). Biogeographic characterization of blue whale song worldwide: using song to identify populations. J. Cetacean. Res Manage.

[22] Oleson EM (2007). Behavioral context of call production by eastern North Pacific blue whales. Mar. Ecol. Prog. Ser..

[23] McDonald MA, Calambokidis J, Teranishi AM, Hildebrand JA (2001). The acoustic calls of blue whales off California with gender data. J Acoust Soc Am..

[24] Oleson EM, Wiggins SM, Hildebrand JA (2007). Temporal separation of blue whale call types on a Southern California feeding ground. Anim Behav..

[25] Nelson, R. J. & Kriefsfeld, L. J. An Introduction to Behavioral Endocrinology. 5th ed (Sinauer Associates, Sunderland, 2016).

[26] Bauer, S. *et al*. Cues and decision rules in animal migration. In: (eds.) E. J. Milner-Gulland, J. M. Fryxell, A. R. E. Sinclair. Animal Migration – A Synthesis (Oxford University Press, Oxford, 2011).

[27] Sherrill-Mix SA, James MC, Myers RA (2007). Migration cues and timing in leatherback sea turtles. Behav Ecol..

[28] Sims DW, Wearmouth VJ, Genner MJ, Southward AJ, Hawkins SJ (2004). Low-temperature-driven early spawning migration of a temperate marine fish. J Anim Ecol.

[29] Nishiwaki, M. Ryukyuan humpback whaling In: Scientific Reports to the Whales Research Institute **15**, 1-15 (1960).

[30] Visser F, Hartman KL, Pierce GJ, Valavanis VD, Huisman J (2011). Timing of migratory baleen whales at the Azores in relation to the North Atlantic spring bloom. Mar. Ecol. Prog. Ser..

[31] Tsujii K (2016). The migration of fin whales into the southern Chukchi Sea as monitored with passive acoustics. CES J. Mar. Sci..

[32] Bracis C, Mueller T (2017). 2Memory, not just perception, plays an important role in terrestrial mammalian migration. Proc. R. Soc. B.

[33] Mettke-Hofmann C, Gwinner E (2003). Long-term memory for a life on the move. PNAS.

[34] Abrahms B (2019). Memory and resource tracking drive blue whale migrations. PNAS.

[35] Ju S, Kang H, Kim WS, Harvey HR (2009). Comparative lipid dynamics of euphausiids from the Antarctic and Northeast Pacific Oceans. Mar. Biol..

[36] Brinton E (1962). The distribution of Pacific euphausiids. Bull. Scripps Inst. Oceanogr..

[37] Lee RF, Hirota J, Barnett AM (1971). Distribution and importance of wax esters in marine copepods and other zooplankton. Deep-Sea Res. Oceanogr. Abstr..

[38] Brinton E (1979). Parameters relating to the distributions of planktonic organisms, especially euphausiids in the eastern tropical Pacific. Prog. Oceanogr..

[39] Gendron D (1992). Population structure of daytime surface swarms of Nyctiphanes simplex (Crustacea: Euphausiacea) in the Gulf of California, Mexico. Mar. Ecol. Prog. Ser..

[40] Reilly SB, Thayer VG (1990). Blue whale (*Balaenoptera musculus*) distribution in the eastern tropical Pacific. Mar Mam Sci.

[41] Paniagua-Mendoza A, Gendron D, Romero-Vivas E, Hildebrand JA (2017). Seasonal acoustic behavior of blue whales (*Balaenoptera musculus*) in the Gulf of California, Mexico. Mar Mam Sci.

[42] Stimpert AK, Peavey LE, Friedlaender AS, Nowacek DP (2012). Humpback whale song and foraging behavior on an Antarctic feeding ground. PLoS One.

[43] Vu ET (2012). Humpback whale song occurs extensively on feeding grounds in the western North Atlantic Ocean. Aquat. Biol.

[44] Herman LM (2017). The multiple functions of male song within the humpback whale (Megaptera novaeangliae) mating system: review, evaluation, and synthesis. Biol. Rev..

[45] Payne R, Webb D (1971). Orientation by means of long range acoustical signaling in baleen whales. Ann. N. Y. Acad. Sci..

[46] Parmesan C, Yohe G (2003). A globally coherent fingerprint of climate change impacts across natural systems. Nature.

[47] Ramp C, Delarue J, Palsbøll PJ, Sears R, Hammond PS (2015). Adapting to a Warmer Ocean - Seasonal Shift of Baleen Whale Movements over Three Decades. PLoS ONE.

[48] Hauser DDW (2016). Decadal shifts in autumn migration timing by Pacific Arctic beluga whales are related to delayed annual sea ice formation. Glob. Change Biol..

[49] Lilly LE, Ohman MD (2018). CCE IV: El Niño-related zooplankton variability in the southern California Current System. Deep Sea Res..

[50] Poloczanska ES (2016). Responses of Marine Organisms to Climate Change across Oceans. Front Mar Sci.

[51] Braithwaite JE, Meeuwig JJ, Hipsey MR (2015). Optimal migration energetics of humpback whales and the implications of disturbance. Conserv. Physiol..

[52] Silva MA, Prieto R, Jonsen I, Baumgartner MF, Santos RS (2013). North Atlantic Blue and Fin Whales Suspend Their Spring Migration to Forage in Middle Latitudes: Building up Energy Reserves for the Journey?. PLoS ONE.

[53] Berman-Kowalewski M (2010). Association between blue whale mortality and ship strikes along the California coast. Aquat Mamm..

[54] Hunt KE (2017). Multiple steroid and thyroid hormones detected in baleen from eight whale species. Conserv. Physiol..

[55] Ball HC (2017). Beyond thermoregulation: metabolic function of cetacean blubber in migrating bowhead and beluga whales. J. Comp. Physiol. B.

[56] Zieba DA, Amstalden M, Williams GL (2005). Regulatory roles of leptin in reproduction and metabolism: a comparative review. Domest Anim Endocrinol.

[57] Hunt KE (2018). Multi-year patterns in testosterone, cortisol and corticosterone in baleen from adult males of three whale species. Conserv. Physiol..

[58] Vu ET, Clark C, Catelani K, Kelllar NM, Calambokidis J (2015). Seasonal blubber testosterone concentrations of male humpback whales (*Megaptera novaeangliae*). Mar Mam Sci.

[59] Cates KA, Atkinson S, Gabriele CM, Pack AA, Straley JM (2019). Testosterone trends within and across seasons in male humpback whales (*Megaptera novaeangliae*) from Hawaii and Alaska. Gen. Comp. Endocrinol..

[60] Wiggins, S.M. & Hildebrand, J.A. High-frequency acoustic recording package (HARP) for broad-band, long-term marine mammal monitoring. In International Symposium on Underwater Technology 2007 and International Workshop on Scientific Use of Submarine Cables & Related Technologies 2007 (Institute of Electrical and Electronics Engineers, Tokyo, Japan, 2007).

[61] Wiggins SM (2010). TRITON software package: Analyzing large passive acoustic monitoring data sets using MATLAB. J Acoust Soc Am..

[62] Helble TA, Ierley G, D’Spain GL, Roche MA, Hildebrand JA (2012). A generalized power-law detection algorithm for humpback whale vocalizations. J Acoust Soc Am..

[63] Mellinger D, Clark C (2000). Recognizing transient low-frequency whale sounds by spectrogram correlation. J Acoust Soc Am..

[64] Širović A (2015). Seven years of blue and fin whale call abundance in Southern California. Endanger. Species Res..

[65] Lavaniegos BE, Ohman MD (2007). Coherence of long-term variations of zooplankton in two sectors of the California Current System. Prog Oceanogr.

[66] Brinton E (1967). Vertical migration and avoidance capability of euphausiids in the California current. Limnol Oceanogr.

[67] Akaike, H. Prediction and Entropy In: (eds.) A.C. Atkinson, SE Fienberg. A celebration of statistics (Springer Verlag, New York, 1985).

[68] Amrhein V, Greenland S, McShance B (2019). Scientists rise up against statistical significance. Nature..

