# Supplementary material for: Timing is everything: Drivers of interannual variability in blue whale migration
Source: Sci Rep. 2020 May 7;10:7710. doi: 10.1038/s41598-020-64855-y (PMC7206123; doi:10.1038/s41598-020-64855-y)
Supplement: Supplementary file 1 — Supplementary Information. [file 41598_2020_64855_MOESM1_ESM.docx]

**Timing is everything: Drivers of interannual variability in blue whale migration**

**Supplementary Materials**

Angela R. Szesciorka^a*^, Lisa T. Ballance^a,b,c^, Ana Širović^d^, Ally Rice^a^, Mark D. Ohman^a^, John A. Hildebrand^a^, Peter J.S. Franks^a^

^a^Scripps Institution of Oceanography, UC San Diego, 9500 Gilman Dr., La Jolla, CA 92093

^b^Southwest Fisheries Science Center, NOAA Fisheries Service, 8901 La Jolla Shores Dr., La Jolla, CA 92037

^c^Oregon State University, Marine Mammal Institute, 2030 SE Marine Science Dr., Newport, Oregon 97365

^d^Texas A&M University at Galveston, 200 Seawolf Parkway, Galveston, TX 77554

^*^corresponding author (angela@szesciorka.com)

**Supplementary Tables**

**Supplementary Table 1**. D and B call migration metrics from 2008 to 2017, including Julian date of onset, peak, cessation and duration. Onset and cessation date of each call type were calculated as thresholds that encompass 90% of the calls relative to the day with the peak number of calls.

**Supplementary Table 2**. High-frequency acoustic recording package (HARP) deployment information, including site, deployment number, start and end datetime, location, depth (m), sample rate, duty cycle, and data start and end datetime.

**Supplementary Table 3. Environmental indices used in the multivariate linear regression modeling.** The two Pacific Ocean basin indices include North Pacific Gyre Oscillation (NPGO) and the Pacific Decadal Oscillation (PDO). The Equatorial index is the Oceanic Niño Index (ONI). The regional environmental indices included sea surface temperature anomalies (SSTs) and chlorophyll *a* (Chl *a*) in the Southern California Region (SCR) and Costa Rica Dome (CRD). The other two SCR environmental indices included the cumulative upwelling index (CUI) and spring adult and juvenile *Euphausia pacifica* and *Thysanoessa spinifera* biomass (Krill).

**Supplementary Table 4**. California Cooperative Oceanic Fisheries Investigations (CalCOFI) cruise information, including cruise number, ship name, dates, lines, and total number of stations from 2008 to 2017 where adult and juvenile *Euphausia pacifica* and *Thysanoessa spinifera* biomass were enumerated (mg Carbon per m^2^) from night tows at lines 80-93 and stations 26-60 (see Supplementary Fig. S6).

**Supplementary Table 5**. Stepwise multiple regression with Akaike information criterion (AIC) for estimating factors acting on D call onset. Adjusted R^2^ for full model: 0.9178. Significant denoted with asterisks.

**Supplementary Figures**


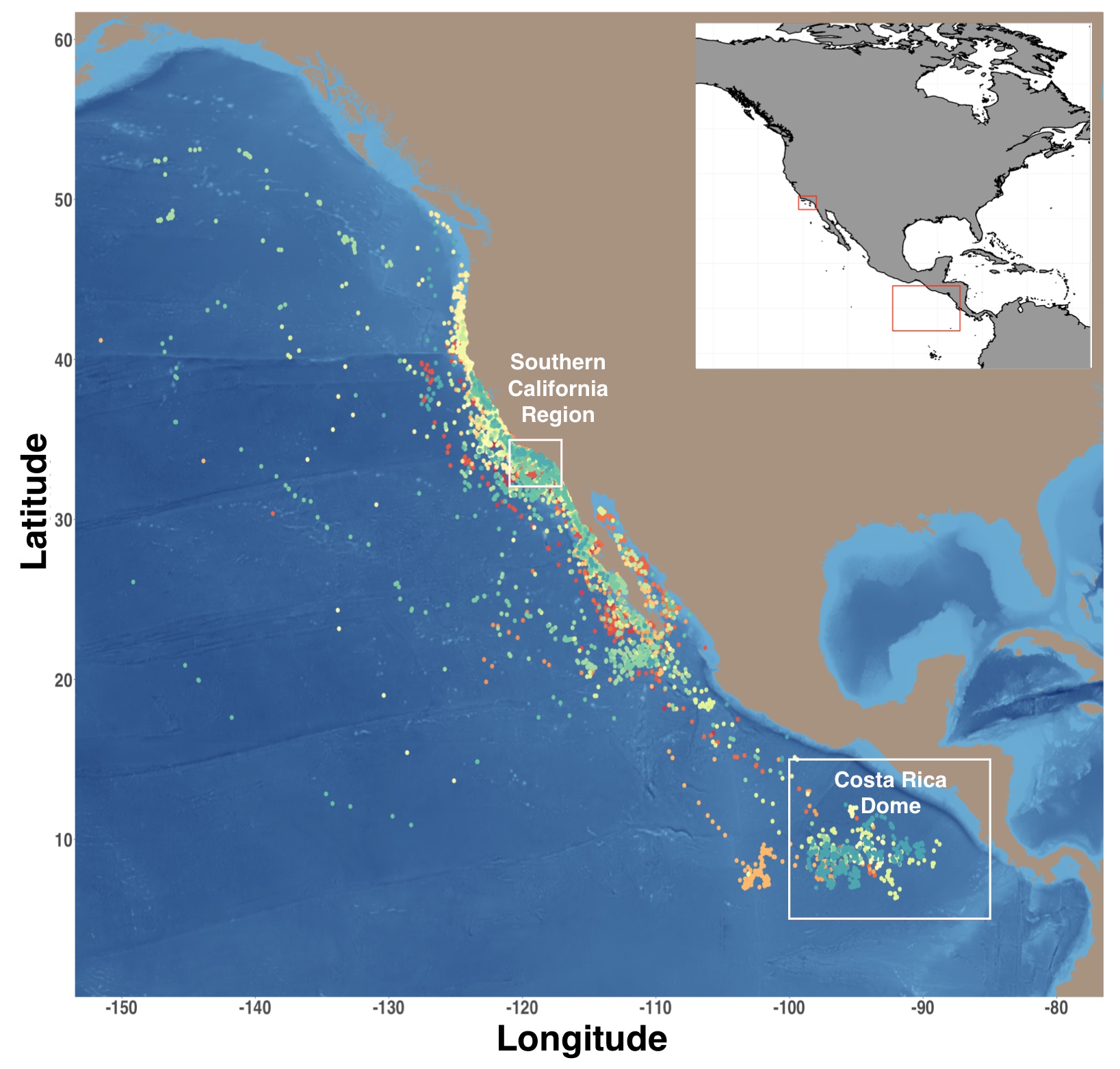


**Supplementary Figure 1**. GPS positions from satellite tagged blue whales (n=122) showing movement between summer feeding grounds in and north of the Southern California Region (32-35N, 121-117W) and winter breeding grounds in the Costa Rica Dome (5-15°N, 100-85°W). Colors correspond with individual whales. Only Argos location classes with accuracy estimations were plotted. Tag locations included Northern California (n=22), Central California (n=17), Southern California (n=78), Baja California (n=3), and the Costa Rica Dome (n=2). Tagging dates spanned 1993–2008, and tags remained attached from 1 to 504 days with an average of 95 days. The bounding boxes were also used for 8-day area-averaged environmental indices derived from satellite imagery, including sea surface temperature (night only; °C) and chlorophyll (mg/m^3^) from MODIS-Aqua level-3 data. Telemetry data downloaded from Movebank’s data repository.^1,2^ Bathymetry data came from the marmap package (v1.03, https://github.com/ericpante/marmap)^3^ in R.^4^ Land polygons were made with Natural Earth (v4.1.1, naturalearthdata.com) in R.^4^


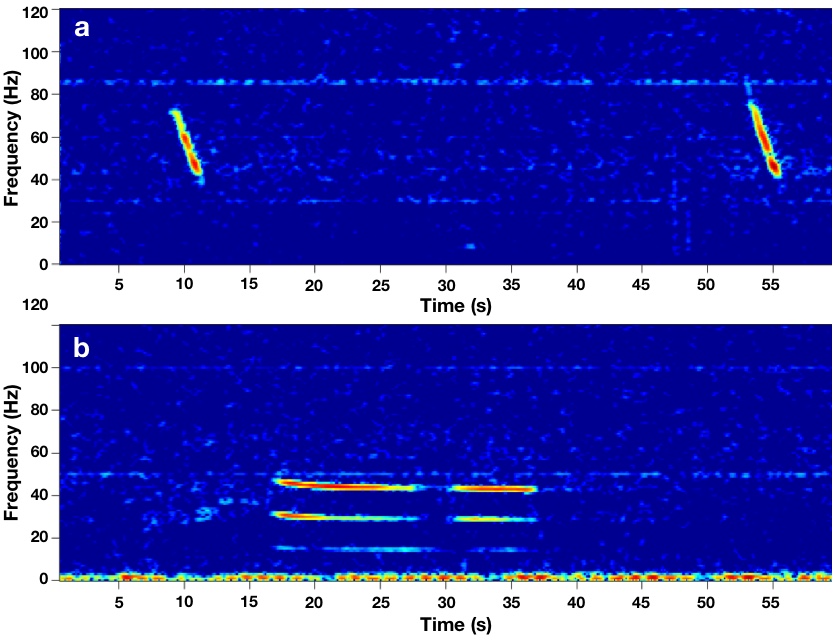


**Supplementary Figure 2**. Spectrograms of (a) two D calls recorded at site B on April 11, 2015 00:45:12 UTC and (b) one B call recorded at the same location on September 1, 2015 00:42:42 UTC. Spectrogram created with 2000-point fast Fourier Transform and 95% overlap, with Hanning window.

**
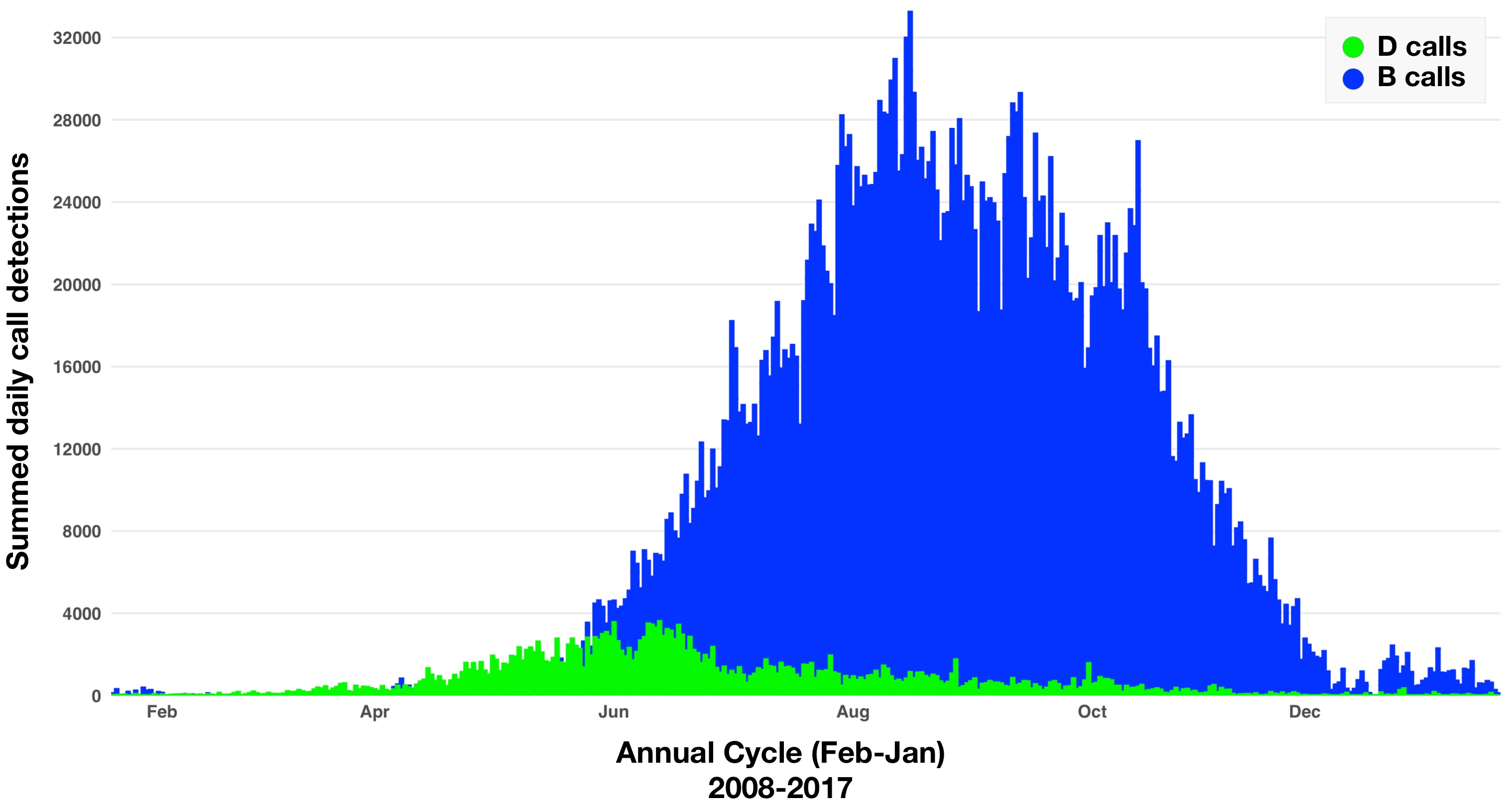
**

**Supplementary Figure 3**. The canonical distribution of daily D and B call detections (combined from all sites and all years) showing the temporal separation between two call types recorded on high-frequency acoustic recording package (HARP) deployment at five sites (see Fig. S5) from 2008 to 2017 in the Southern California Region.

**
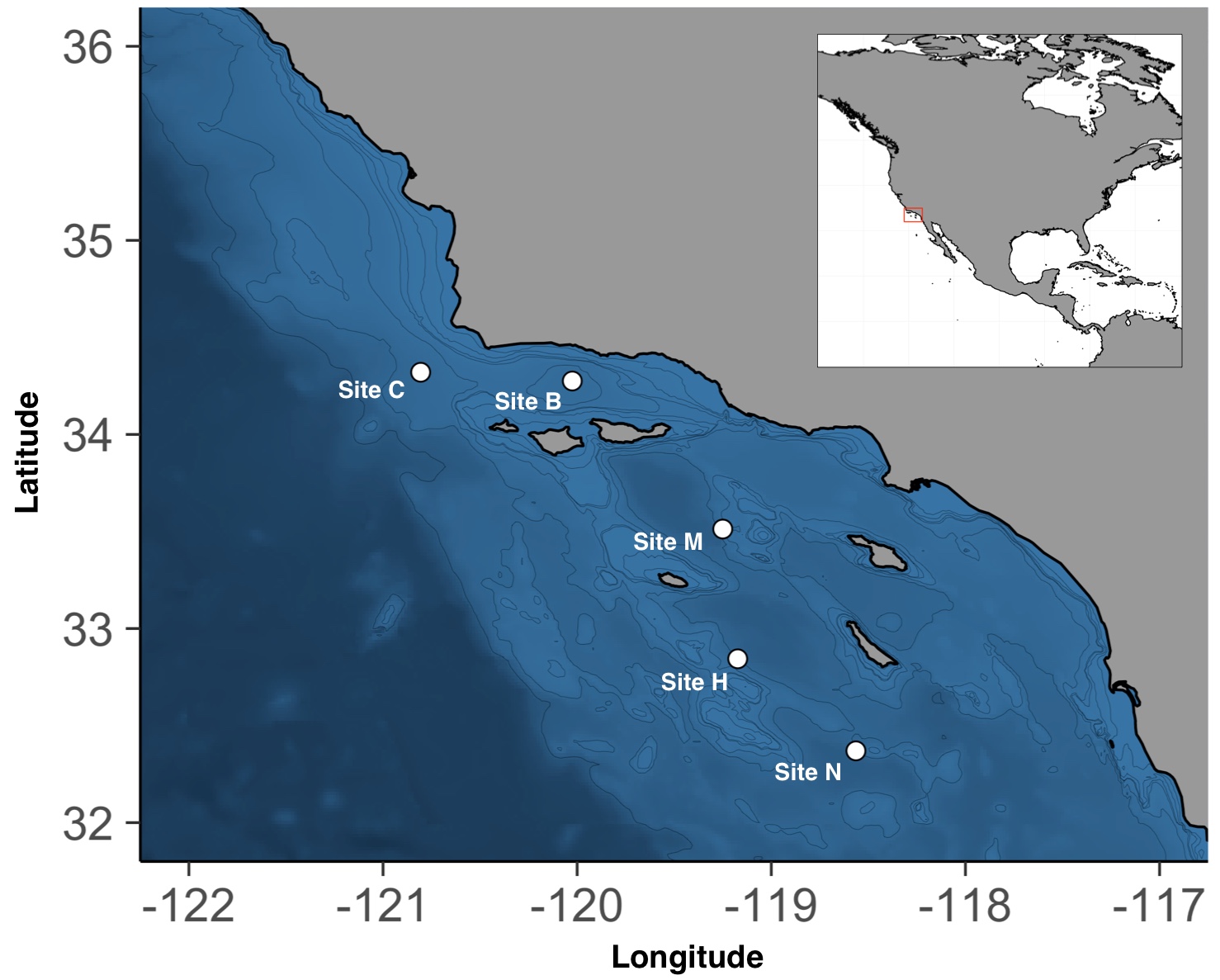
**

**Supplementary Figure 4.** Five high-frequency acoustic recording package (HARP) deployment sites (white circles) from 2008 to 2017 in the Southern California Region (see inset box map), including sites B, C, H, M, and N. Bathymetry data came from the marmap package (v1.03, https://github.com/ericpante/marmap)^3^ in R.^4^ Land polygons were made with Natural Earth (v4.1.1, naturalearthdata.com) in R.^4^

**
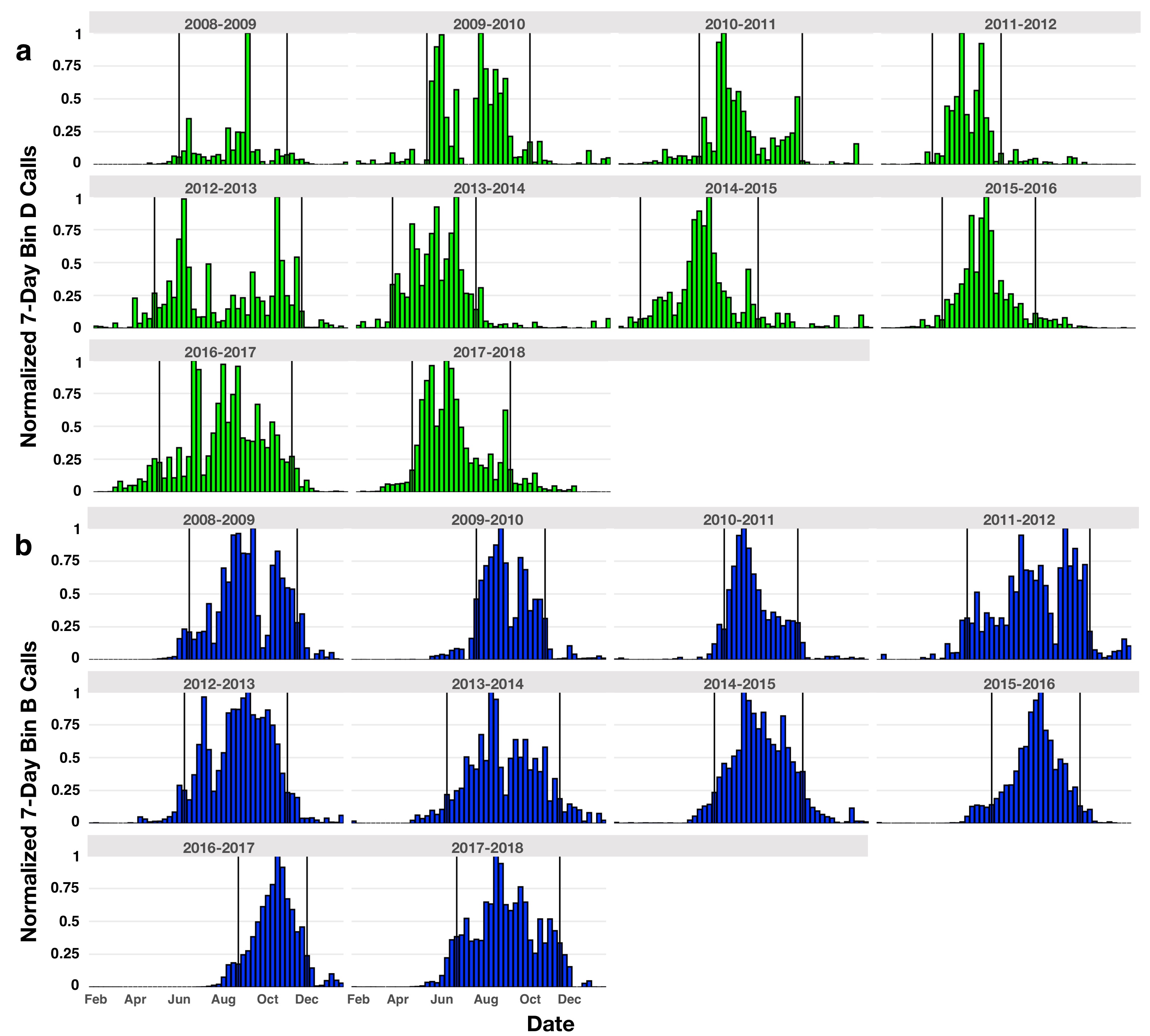
**

**Supplementary Figure 5**. Annual distribution of for (a) D calls and (b) B calls from 2008 to 2017. Calls have been pooled across sites, binned into weekly medians, and normalized to be between 0 and 1 by scaling with the maximum number of calls per annual cycle. The black bars around each annual cycle indicate the call cutoffs, which encompass 90% of calls from the day with the peak number of calls.


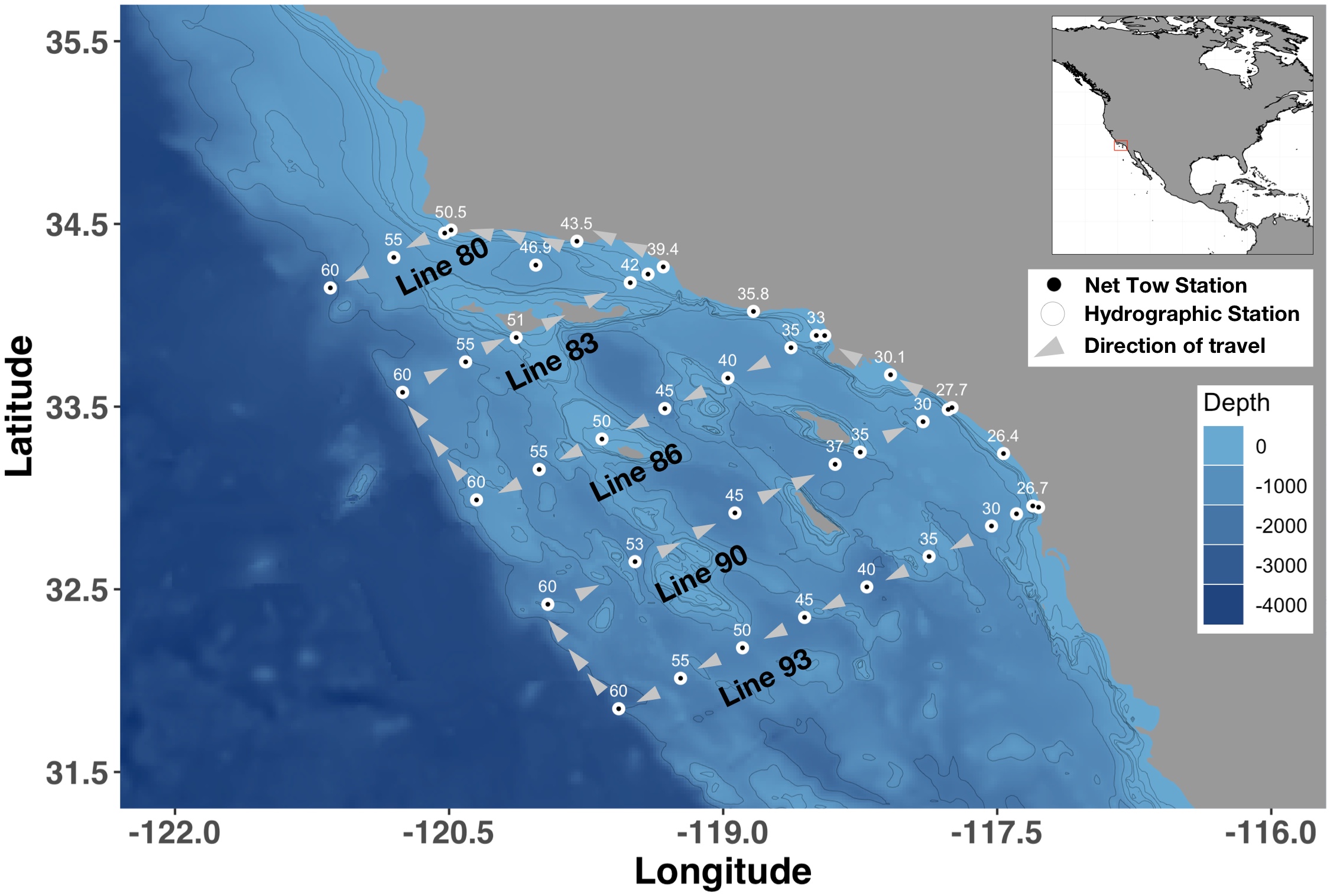


**Supplementary Figure 6**. Location of bongo net tow and hydrographic sampling stations and direction of travel during springtime California Cooperative Oceanic Fisheries Investigations cruises in the Southern California Region from 2008 to 2017, where adult and juvenile *Euphausia pacifica* and *Thysanoessa spinifera* were collected from night tows at lines 80-93 and stations 26-60. Bathymetry data came from the marmap package (v1.03, https://github.com/ericpante/marmap)^3^ in R.^4^ Land polygons were made with Natural Earth (v4.1.1, naturalearthdata.com) in R.^4^


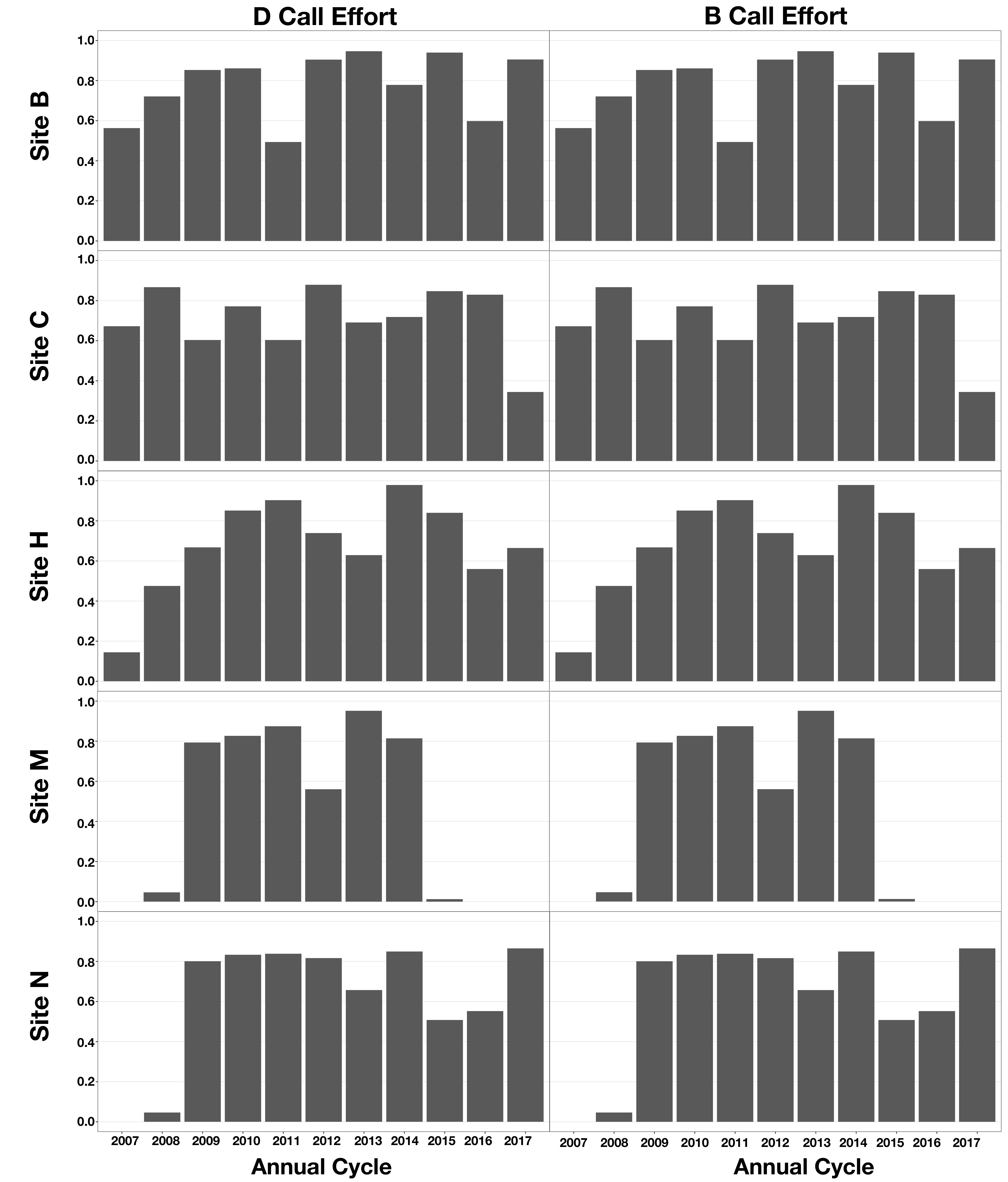


**Supplementary Figure 7**. Acoustic recording effort (as a fraction from 0 to 1) for D and B calls by site for D calls (left panel) and B calls (right panel).

**Supplementary Methods and Analyses**

*Using calls as a proxy for presence/absence*

We used the presence of calls as a proxy for the presence of blue whales in the SCR, ultimately to determine their arrival and departure dates. While the absence of calls recorded on the HARPs could be indicative of whale absence on the feeding grounds, it could also indicate the presence of silent whales. However, the goal for this study and impetus behind our call threshold method was to examine the main period of presence. Historic whaling records^5^, visual sighting data^6^, satellite tags^1,2^ , and acoustic recordings^7,8^ all result in similar estimates of blue whale presence in this area, so our method is likely capturing presence well.

*Call pooling*

To ensure our pooling method for the calls did not create a bias, we compared the weekly binned medians that we used in this study with other pooling methods, including bin sizes at 5, 7, 10, and 14 days. We also tested various weekly binning methods, including summation, site-specific maximums, site-specific medians, and sites binned separately and then combined. In all comparisons, the relationship between the call metrics (i.e., onset, peak, cessation, duration) was comparable, and plotted with roughly one-to-one slope. This suggested that our method for pooling calls would result in a similar outcome as any other method for pooling the calls.

*Call normalizing*

In this study we were not interested in the total number of calls or quantifying the density of animals present in the SCR. There was also no way to account for whale movement among HARP sites, which could result in double counting. Because we were concerned only with the timing of whales’ presence in the SCR, the weekly binned medians were normalized to be between 0 and 1 by scaling with the maximum number of calls per annual cycle. This gave us relative values that we could use to determine when blue whales were present in the Southern California Region.

*Call cutoffs*

We tested for differences in call cut-offs for defining onset and cessation for each call. There was no significant difference in 0.85, 0.90, or 0.95 percent call cut-offs. We chose a cutoff of 90 percent so that we would only eliminate five percent of calls either side from the day with the peak number of calls.

*HARP effort differences*

There were differences among HARP sites both in terms of recording effort and overall patterns in D and B call occurrence (Fig. S7). However, there were no significant differences in the number of HARPs operating the month of each call metric, or in the month preceding call onset dates and following call cessation dates. The differences in call occurrence across site are also due to difference in whale distribution in the area, but differences in detection probability could also play a role.^9^ Comparing the location of HARP sites to the predicted density of blue whales in the Southern California Region^10^, no site stood out as having a greater predicted density relative to other sites. There could also have been annual variability in the recording quality at any one site, which could result in systemic bias in a portion of the data. However, by combining all sites we believe we have captured the general pattern of blue whale presence in the Southern California Region.

*Additional upwelling indices*

To best capture the phenology of environmental conditions in the SCR, we also investigated the relationship between call metrics and the Coastal Upwelling Transport Index (CUTI) and the Biologically Effective Upwelling Transport Index (BEUTI). CUTI provides estimates of vertical transport near the coast (i.e., upwelling or downwelling), while BEUTI provides estimates of vertical nitrate flux near the coast (i.e., amount of nitrate upwelled or downwelled).^11^ The lags matched the lag from cumulative upwelling index in the SCR and thus did not add any additional information about blue whale migration timing.

**Supplementary References**

1. Bailey, H., *et al*. Blue whale behavior in the eastern North Pacific inferred from state-space model analysis of satellite tracks. *Endanger. Species Res.* **10,** 93-106 (2009).
2. Mate, B., Lagerquist, B., Calambokidis, J. Movements of North Pacific blue whales during the feeding season off southern California and their southern fall migration. *Mar Mam Sci* **15,** 1246-1257 (1999).
3. Pante, E., Simon-Bouhet, B. marmap: A Package for Importing, Plotting and Analyzing Bathymetric and Topographic Data in R. *PLoS ONE* **8(9),** e73051 (2013).
4. R Core Team. R: A language and environment for statistical computing. R Foundation for Statistical Computing, Vienna, Austria. https://www.R-project.org/ (2020).
5. Scammon, C.M. The marine mammals of the north-western coast of North America and the American whale fishery (G.P. Putnam’s Sons, New York, 1874).
6. Calambokidis, J., *et al*. Sightings and movements of blue whales off central California 1986–88 from photo‐identification of individuals. *Rep. int. Whaling Comm.* (Special Issue) **12,** 343-348 (1990).
7. Stafford, K.M., Nieukirk, S.L., Fox, C.G. Low frequency whale sounds recorded on hydrophones moored in the eastern tropical Pacific. *J Acoust Soc Am.* **106**, 3687-3698 (1999).
8. Burtenshaw, J.C., *et al*. Acoustic and satellite remote sensing of blue whale seasonality and habitat in the Northeast Pacific. *Deep Sea Res. Pt II* **51,** 967-986 (2004).
9. Širović, A., *et al*. Seven years of blue and fin whale call abundance in southern California. *Endanger. Species Res.* **28,** 61-76 (2015).
10. Redfern, J.V., *et al*. Assessing the risk of chronic shipping noise to baleen whales off Southern California, USA. *Endanger. Species Res.* **32,** 153-167 (2017).
11. Jacox, M.G., Edwards, C.A., Hazen, E.L., Bograd, S.J. Coastal upwelling revisited: Ekman, Bakun, and Improved Upwelling Indices for the U.S. West Coast. *J. Geophys Res. Oceans* **123(10),** 7332-7350 (2018).
